# DPP4/CD26 overexpression in urothelial carcinoma confers an independent prognostic impact and correlates with intrinsic biological aggressiveness

**DOI:** 10.18632/oncotarget.13820

**Published:** 2016-12-07

**Authors:** Peir-In Liang, Bi-Wen Yeh, Wei-Ming Li, Ti-Chun Chan, I-Wei Chang, Chun-Nung Huang, Ching-Chia Li, Hung-Lung Ke, Hsin-Chih Yeh, Wen-Jeng Wu, Chien-Feng Li

**Affiliations:** ^1^ Department of Pathology, Kaohsiung Medical University Hospital, Kaohsiung Medical University, Kaohsiung, Taiwan; ^2^ Department of Urology, Kaohsiung Medical University Hospital, Kaohsiung, Taiwan; ^3^ Department of Urology, School of Medicine, College of Medicine, Kaohsiung, Medical University, Kaohsiung, Taiwan; ^4^ Graduate Institute of Medicine, College of Medicine, Kaohsiung Medical University, Kaohsiung, Taiwan; ^5^ Department of Urology, Ministry of Health and Welfare Pingtung Hospital, Pingtung, Taiwan; ^6^ Department of Medical Research, Chi Mei Medical Center, Tainan, Taiwan; ^7^ Institute of Biomedical Science, National Sun Yat-sen University, Kaohsiung, Taiwan; ^8^ Department of Pathology, E-DA Hospital, I-Shou University, Kaohsiung, Taiwan; ^9^ School of Medicine for International Students, I-Shou University, Kaohsiung, Taiwan; ^10^ Department of Urology, Kaohsiung Municipal Ta-Tung Hospital, Kaohsiung, Taiwan; ^11^ Center for Infectious Disease and Cancer Research, Kaohsiung Medical University, Kaohsiung, Taiwan; ^12^ Center for Stem Cell Research, Kaohsiung Medical University, Kaohsiung, Taiwan; ^13^ Institute of Medical Science and Technology, National Sun Yat-sen University, Kaohsiung, Taiwan; ^14^ Department of Pathology, Chi Mei Medical Center, Tainan, Taiwan; ^15^ Department of Biotechnology, Southern Taiwan University of Science and Technology, Tainan, Taiwan; ^16^ National Institute of Cancer Research, National Health Research Institutes, Tainan, Taiwan; ^17^ Institute of Clinical Medicine, Kaohsiung Medical University, Kaohsiung, Taiwan

**Keywords:** urothelial carcinoma, DPP4, overexpression, proteolysis

## Abstract

Urothelial carcinoma (UC) is common cancer worldwide. The molecular aberrations regarding tumor progression remain unclear. Pericellular proteolysis is crucial in tumorigenesis, but its significance is unexplored in UC. By data mining the datasets in Gene Expression Omnibus, specifically focus on the proteolysis pathway, and followed by a preliminary validation in a pilot batch of tumor samples, we identified that the upregulation of *dipeptidyl peptidase 4 (DPP4)* was most significantly associated with clinical aggressiveness of UCs. Quantitative RT-PCR confirmed upregulation of *DPP4* mRNA in advanced stage UCs. The clinical significance of DPP4 expression was validated in our large cohort consists of 635 UCs from upper urinary tract and urinary bladder. Univariate and multivariate analyses show that DPP4 is an independent prognosticatory biomarker for disease-specific survival and metastasis-free survival. Comparing the DPP4 expression level of three urothelial cell lines with normal urothelial cells, J82 and RTCC-1 showed a significantly increased in transcript and protein expression. DPP4 knockdown as conducted by using short-hairpin RNA resulted in a significantly decreased cell viability, proliferation, migration, and invasion in J82 and RTCC-1 cells. These findings implicate that DPP4 plays a role in the aggressiveness of UCs, and can serve as a novel prognostic marker and therapeutic target.

## INTRODUCTION

Urothelial carcinoma (UC) is common cancer worldwide that arises from both the upper urinary tract (UUT, renal pelvis, and ureter) and lower urinary tract (LUT, urinary bladder, and urethra). [1] Generally, the incidence of UC of the urinary bladder (UBUC) is more frequent than UC of the upper urinary tract (UTUC); the ratio of the incidence of urothelial carcinoma in the renal pelvis, ureter, and urinary bladder is approximately 3:1:51. [2] The etiology of UC, regardless of location, includes smoking cigarettes and exposure to aromatic amines containing chemicals. However, certain etiologies are more common in patients with UTUC, such as Balkans endemic nephropathy, Chinese herb nephropathy, and phenacetin abuse. [3] Nevertheless, the disease behavior of stage-adjusted UTUC and UBUC is identical, and the gene expression profiles of UCs from both locations are very similar. [3, 4] This may indicate that tumorigenesis of UC arising at any site in the urinary tract shares a common pathway. Although there is an increasing number of biomarkers that are prognostic relevant to UCs, factors regarding tumor progression remained largely unclear [5].

Carcinogenesis of human cancers is a multi-step process that eventually transforms a normal cell into a malignant neoplasm [6]. The interaction between the tumor cells and the microenvironment is an important event during tumorigenesis. Dysregulated proteolysis has long been linked to cancer development. In fact, the increase of proteases production has been reported in various cancers and is often associated with poor outcome [7].

By mining the datasets obtained from the Gene Expression Omnibus (GEO, NCBI, Bethesda, MD, USA) and focused on the proteolysis pathway, we discovered that the transcription of *dipeptidyl peptidase 4* (*DPP4*) was significantly upregulated in advanced-stage human urothelial carcinomas. DPP4, also known as CD26, is an 110 kDa transmembrane glycoprotein that encoded by a gene located at chromosome 2q23. It belongs to the DPP4 family, an ubiquitously expressed serine peptidase family. DPP4/CD26 can presence as a membrane bound protein or as a soluble form enzyme (sCD26) [8]. It functions as an ectopeptidase that can inactivate incretins, cleavage of chemokines, promote cell migration, activation of lymphocytes, etc [9]. The expression of DPP4 is linked to the carcinogenesis of many malignant tumors [10].

The role of DPP4 in tumorigenicity is variable in different tumors [8]. In some tumors, such as astrocytoma, gastrointestinal stromal tumors, and some lymphomas, overexpression of DPP4 is associated with tumor aggressiveness [11–15]. On the other hand, the absence or loss of expression of DPP4 is observed in the advanced stage of certain malignancies, including melanomas, endometrial carcinoma, and lung squamous cell carcinoma [16–20]. To our knowledge, the association between DPP4 expression and UC has never been evaluated. Therefore, we set out to systematically analyze the impact of DPP4 expression on the clinical and pathological behavior of UCs and to assess the function of DPP4 in the tumorigenesis of the urothelial cancer cell.

## RESULTS

### DPP4 was identified as a significant differentially upregulated transcript in UBUC

**Figure 1:**
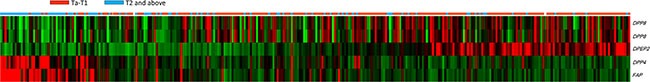
Data mining on GSE32894 (GEO omnibus) dataset identified four transcripts (5 probes) that were significantly associated with proteolysis (GO: 0006508) in urinary bladder urothelial carcinoma (UBUC) A heat map of specimen with low (n = 215) and high (n = 93) pT are shown. Low expression values are green, progression into dark and reds for higher values. The transcriptomes of 308 cases of UBUCs reconstructed from GSE32894 showed that up-regulation of DPP4 and FAP expression are associated with the advanced pT stage. DPP8 (2 probes) and DPEP2 expression are inversely associated with the pT stage.

**Figure 2:**
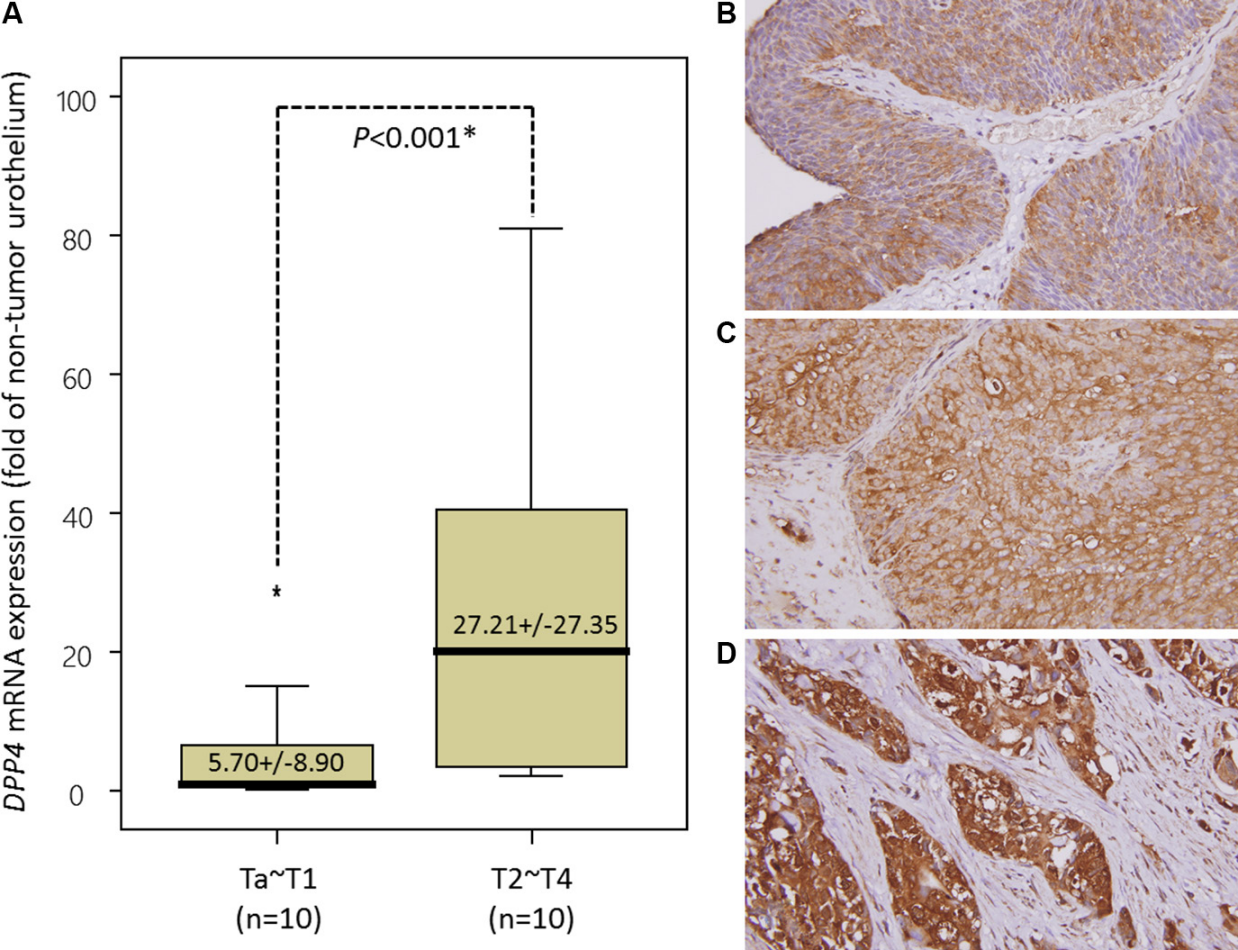
Validation of DPP4 mRNA level and DPP4 protein expression in urinary carcinoma (UC) specimens (**A**) To validate DPP4 transcript expression, we measure the mRNA level of DPP4 in 20 snap frozen UBUC specimens by using laser capture microdissection coupled with real-time quantitative RT-PCR. DPP4 mRNA level was significantly increased in UBUC of advanced stage (pT2-pT4). (**B–D**) The expression of DPP4 protein was further tested in a larger cohort of patients, consists of 295 UBUCs and 340 UTUCs. Low-grade and non-invasive urothelial cancer (B) shows very low level of DPP4 expression in the membrane and cytoplasm. The DPP4 immunoreactivity is significantly elevated in superficially invasive urothelial carcinoma (C) and is more diffuse and stronger in high-grade and muscle-invasive invasive urothelial carcinoma (D). (Magnification, 200×).

From the transcriptomic profiles of the GSE32894 dataset, we identified five probes covering four transcripts that were associated with regulation of proteolysis (GO:0006508) (Figure 1, Supplementary Table S1). Similar results were also observed by analyzing in the GSE31684 dataset, with four probes covering three transcripts found to be associated (Supplementary Figure S1, Supplementary Table S2). Both *DPP4* and *FAP* that have positive log ratios in both datasets were selected for further study. To evaluate the significance of these two proteins in UCs, a preliminary survey was carried out. An immunohistochemical study using a pilot batch of cases showed that FAP protein was mainly expressed in the stromal cells but not in the tumor cells. Furthermore, FAP expression was not significantly associated with disease-specific survival (DSS) and metastasis-free survival (MeFS) of UC patients (Supplementary Figure S2, Supplementary Table S3). In the other hand, DPP4 expression could be detected in tumor cells and significantly associated with patients DSS and MeFS Supplementary Figure S2A, Figure 2B–2D, Supplementary Table S4. Thus, DPP4 was subjected for further evaluation.

### DPP4 mRNA expression significantly associated with advanced tumor stage

To validate that *DPP4* mRNA expression is significantly associated with higher tumor stage, laser capture microdissection (LCM)-isolated tumor cells from fresh samples of a small cohort of UBUC patients were submitted to quantify the *DPP4* mRNA level. This group composed of 10 patients with early stage tumor (pTa-pT1) and 10 with advanced stage tumor (pT2-pT4). Real-time RT-PCR disclosed that the expression level of *DPP4* mRNA is significantly upregulated in UBUC of advanced stage (*p* < 0.001), in contrast to early stage tumors (Figure 2A).

### DPP4 protein expression in UBUC is correlated with clinical aggressiveness of the disease and worse outcome

To understand the clinical significance of DPP4 expression in UBUC tumors, we evaluated the DPP4 expression in 295 UBUC specimens by using immunohistochemical study. The expression of DPP4 in normal urothelium is low (Figure 2B). The expression of DPP4 is mildly increased in non-invasive or low-grade UC (Figure 2C) but is high in invasive high-grade UC (Figure 2D). The association of DPP4 expression and various clinicopathological factors of our patients are listed in Supplementary Table S5. Majority of UBUC patients were older than 65 years (*n* = 174, 58.9%) and were male (*n* = 216, 73.2%). High expression of DPP4 in UBUC significantly associated with higher tumor pT stage (*p* < 0.001), presence of nodal metastasis (*p* = 0.033), vascular invasion (*p* < 0.001) and perineural invasion (*p* = 0.021).

**Figure 3:**
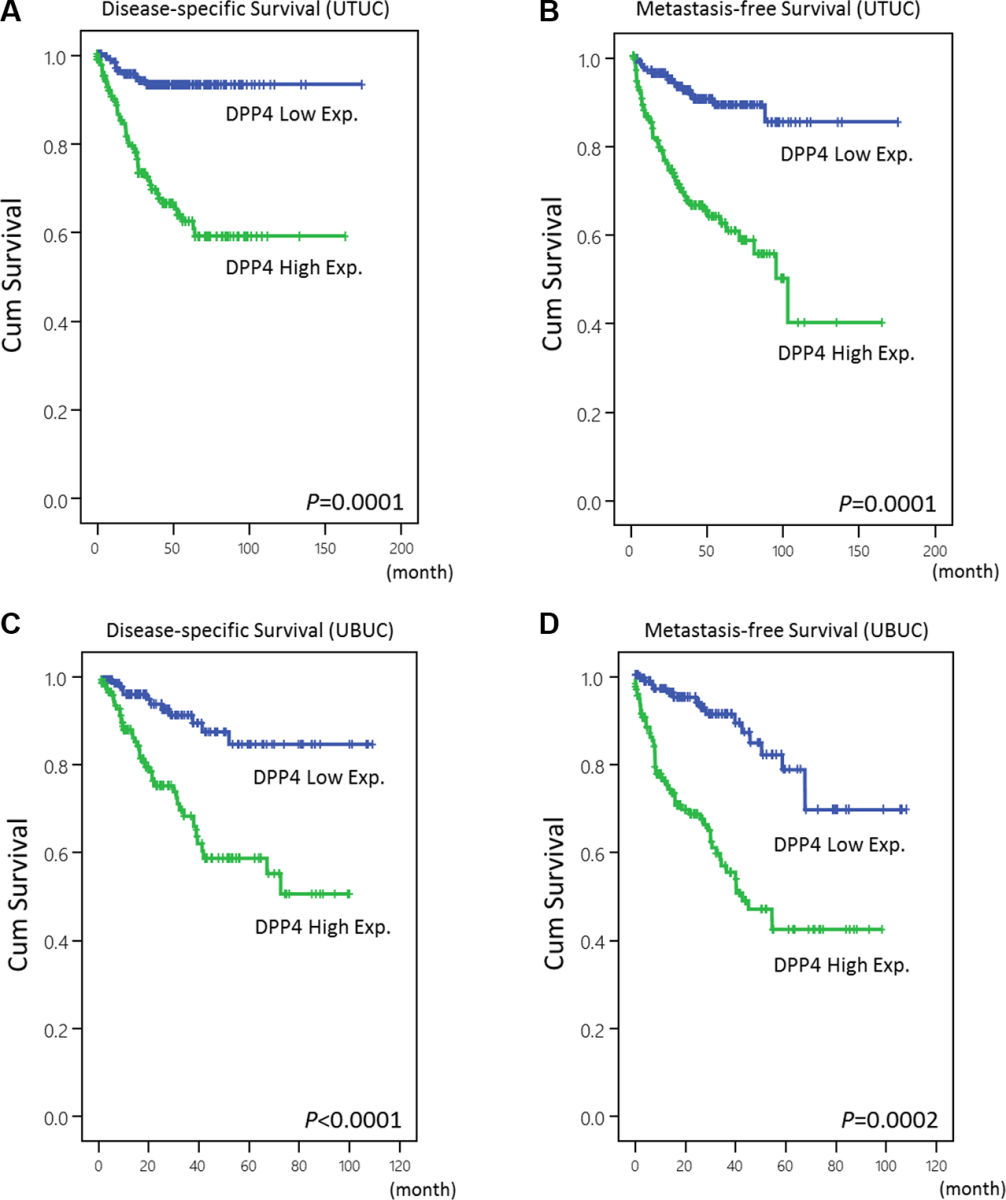
Kaplan-Meier analyses of disease-specific survival (DSS) and metastasis-free survival (MeFS) The plots show that DPP4 overexpression is significantly associated with inferior DSS of UTUC (**A**) and UBUC (**C**). A similar outcome is also demonstrated in MeFS of UTUC (**B**) and UBUC (**D**).

The overexpression of DPP4 in UBUC is correlated with poor DSS (*p* < 0.0001) and MeFS (*p* < 0.0001) (Figure 3, Table 1). Besides, along with primary tumor (pT) stage, perineural invasion, and high mitotic rate, DPP4 expression is an independent prognostic factor of DSS (*p* < 0.001) and MeFS (*p* < 0.001) in UBUC. This finding indicates that DPP4 plays a major role in tumorigenesis of UBUC.

**Table 1:** Univariate log-rank and multivariate analyses for disease-specific and metastasis-free survivals in urinary bladder urothelial carcinoma

Parameter	Category	Case No.	Disease-specific Survival		Metastasis-free Survival	
			Univariate analysis		Multivariate analysis			Univariate analysis		Multivariate analysis		
			No. of event	*p*-value	R.R.	95% C.I.	*p*-value	No. of event	*p*-value	R.R.	95% C.I.	*p*-value
**Gender**	Male	216	41	0.4446	-	-	-	60	0.2720	-	-	-
	Female	79	11		-	-	-	16		-	-	-
**Age (years)**	< 65	121	17	0.1136	-	-	-	31	0.6875	-	-	-
	≥ 65	174	35		-	-	-	45		-	-	-
**Primary tumor (T)**	Ta	84	1	< 0.0001*	1	-	< 0.001*	4	< 0.0001*	1	-	0.004*
	T1	88	9		5.554	0.604–51.101		23		4.513	1.311–15.537	
	T2-T4	123	42		20.442	2.330–179.356		49		6.288	1.835–21.548	
**Nodal metastasis**	Negative (N0)	266	41	0.0002*	1	-	0.217	61	< 0.0001*	1	-	0.025*
	Positive (N1–N2)	29	11		1.552	0.772–3.119		15		**2.003**	**1.091-3.678**	
**Histological**	**grade **	Low grade	56	2	0.0013*	1	-	0.774	5	0.0007*	1	-	0.519
	High grade	239	50		1.249	0.273–5.722		71		1.403	0.502–3.927	
**Vascular invasion**	Absent	246	37	0.0024*	**1**	**-**	0.029*	54	0.0001*	1	-	0.217
	Present	49	15		**2.257**	**1.089–4.695**		22		1.477	0.795–2.747	
**Perineural invasion**	Absent	275	44	0.0001*	**1**	**-**	0.004*	66	0.0007*	**1**	**-**	0.031*
	Present	20	8		**3.748**	**1.518–9.256**		10		**2.352**	**1.081–5.115**	
**Mitotic rate (per 10 high power fields)**	< 10	139	12	< 0.0001*	**1**	**-**	0.010*	23	< 0.0001*	**1**	**-**	0.009*
	> = 10	156	40		**2.398**	**1.230–4.677**		53		**1.978**	**1.187–3.297**	
**DPP4 expression**	Low	147	11	< 0.0001*	**1**	**-**	< 0.001*	17	< 0.0001*	1	-	< 0.001*
	High	148	41		**3.562**	**1.763–7.196**		59		3.530	2.016–6.182	

* Statistically significant.

### DPP4 protein expression in UTUC is correlated with advanced disease and is an independent prognosticatory biomarker

The expression of DPP4 in 340 UTUC specimens was also evaluated to clarify the clinical implication of this protein in the entire spectrum of UCs. In UTUC patients, their age ranged from 34 to 87 (median, 68 years) and the disease showed a slight predilection for females. Overexpression of DPP4 in UTUC correlated with higher tumor pT stage (*p* < 0.001), presence of nodal metastasis (*p* < 0.001), high histological grade (*p* = 0.019), vascular invasion (*p* < 0.001) and frequent mitosis (*p* = 0.003) (Supplementary Table S5).

Univariate analysis shows that high DPP4 expression is associated with dismal DSS (*p* < 0.0001) and MeFS (*p* < 0.0001) outcome in UTUC patients, along with multifocality, primary tumor (pT) stage, nodal metastasis, histological grade, vascular invasion, and perineural invasion (Figure 3, Table 2). Multivariate analysis identified DPP4 expression as one of the independent prognostic factors for DSS (*p* = 0.028) and MeFS (*p* = 0.031) in UTUC.

**Table 2:** Univariate log-rank and multivariate analyses for disease-specific and metastasis-free survivals in upper urinary tract urothelial carcinoma

Parameter	Category	Case No.	Disease-specific Survival		Metastasis-free Survival	
			Univariate analysis		Multivariate analysis			Univariate analysis		Multivariate analysis		
			No. of event	*p*-value	R.R.	95% C.I.	*p*-value	No. of event	*p*-value	R.R.	95% C.I.	*p*-value
**Gender**	Male	158	28	0.8286	-	-	-	32	0.7904	-	-	-
	Female	182	33		-	-	-	38		-	-	-
**Age (years)**	< 65	138	26	0.9943	-	-	-	30	0.8470	-	-	-
	≥ 65	202	35		-	-	-	40		-	-	-
**Tumor side**	Right	177	34	0.7366	-	-	-	38	0.3074	-	-	-
	Left	154	26		-	-	-	32		-	-	-
	Bilateral	9	1		-	-	-	0		-	-	-
**Tumor location**	Renal pelvis	141	24	0.0079*	1	-	0.873	31	0.0659	-	-	-
	Ureter	150	22		0.770	0.413–1.435		25		-	-	-
	Renal pelvis & ureter	49	15		1.331	0.369–4.803		14		-	-	-
**Multifocality**	Single	273	48	0.0026*	**1**	**-**	0.012*	52	0.0127*	**1**	**-**	0.005*
	Multifocal	62	18		**2.658**	**1.241–5.692**		18		**2.201**	**1.288–6.003**	
**Primary tumor (T)**	Ta	89	2	< 0.0001*	**1**	**-**	0.040*	4	< 0.0001*	1	-	0.275
	T1	92	9		**3.562**	**0.757–16.746**		15		2.140	0.143–2.384	
	T2-T4	159	50		**4.004**	**0.869–18.459**		51		3.938	0.047–3.136	
**Nodal metastasis**	Negative (N0)	312	42	< 0.0001*	**1**	**-**	< 0.001*	**55**	< 0.0001*	**1**	**-**	0.001*
	Positive (N1-N2)	28	19		**4.862**	**2.622–9.017**		**15**		**2.711**	**1.465–5.016**	
**Histological grade**	Low grade	56	4	0.0215*	1	-	0.073	3	0.0027*	**1**	**-**	0.034*
	High grade	284	57		2.740	0.912–8.233		67		**3.636**	**1.105–11.965**	
**Vascular invasion**	Absent	234	24	< 0.0001*	1	-	0.316	26	< 0.0001*	**1**	**-**	0.010*
	Present	106	37		1.362	0.745–2.493		44		**2.225**	**1.214–4.075**	
**Perineural invasion**	Absent	321	50	< 0.0001*	1	-	< 0.001*	61	< 0.0001*	**1**	**-**	0.009*
	Present	19	11		**4.049**	**1.924–8.521**		9		**2.705**	**1.282–5.706**	
**Mitotic rate (per 10 high power fields)**		< 10	173	27	0.167	-	-	-	30	**0.0823**	-	-	-
	> = 10	167	34		-	-	-	40		-	-	-
**DPP4 expression**	Low	170	11	< 0.0001*	**1**	**-**	0.028*	12	< 0.0001*	**1**	**-**	0.031*
	High	170	50		**2.383**	**1.099–5.167**		58		**2.048**	**1.068–3.926**	

* Statistically significant.

### DPP4 promotes cell proliferation, migration and invasion ability of UC cell lines

**Figure 4:**
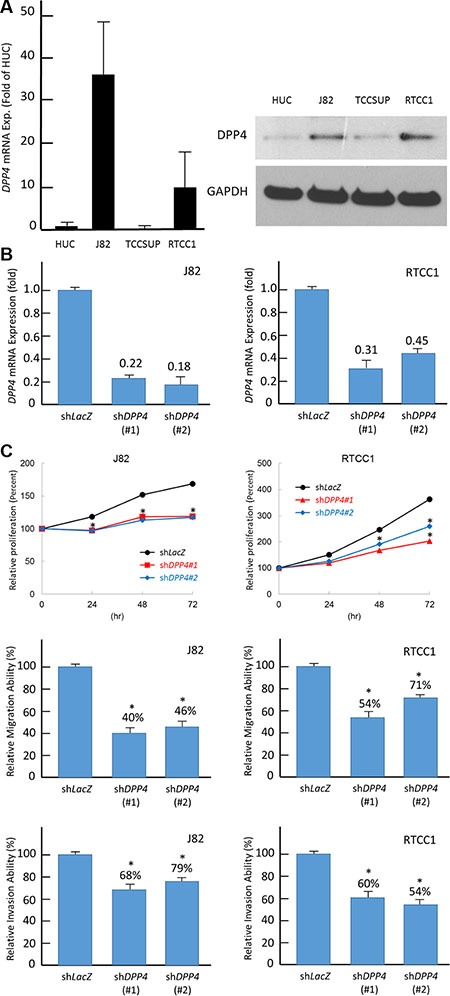
DPP4 expression promotes cell proliferation, migration, and invasion (**A**) Endogenous DPP4 mRNA levels were measured by using quantitative RT-PCR (right panel) and western blotting assays (left panel). Compared with non-tumorigenic urothelial primary cell HUC, two UC cells, J82 and RTCC-1, show high DPP4 mRNA and protein expression levels. (**B**) To explore biological functions of DPP4 in vitro, DPP4 knockdown is conducted by using short-hairpin RNA which successfully deplete DPP4 transcript level in J82 (right panel) and RTCC-1 (left panel) cells. (**C**) Depletion of DPP4 expression results in a significantly decreased cell viability (upper panel), migration (middle panel), and invasion (lower panel) in J82 and RTCC-1 cells. The quantified results are presented as means ± sd. Error bars indicate the standard error of the mean. Data represent mean values of three independent experiments. (*P < 0.05).

To understand the biological function of DPP4, we characterized DPP4 endogenous expression in UC cell lines. In contrast with the normal urothelial primary cell HUC, two out of the three urothelial tumor cell lines, J82 and RTCC-1, showed a significant elevation in *DPP4* mRNA level (Figure 4A). Western blot analysis confirmed that DPP4 protein expression in J82 and RTCC-1 paralleled with the mRNA level (Figure 4A). We then performed DPP4 knockdown in J82 and RTCC-1 cell lines by using two independent clones of shRNA for this gene that successfully depleted DPP4 expression in J82 and RTCC-1 cells (Figure 4B). We first performed flow cytometric and XTT assays to understand if DPP4 regulates UC cell growth. The results show that DPP4 knockdown led to a decreased cell growth by resulting G0/G1 arrest (Figure 4C, Supplementary Figure S3). To clarify if DPP4 promote migration and invasion in UC cell, the DPP4 knockdown UC cells are subjected to migration and invasion assays which demonstrated both the migratory and invasion abilities of the UCs was significantly suppressed after DPP4 knockdown. (Figure 4C) These findings are in concordance with what we have observed that the overexpression of DPP4 is associated with the development of nodal, lymphovascular permeation, perineural invasion, and distal metastasis in the clinical cohort.

### DPP4 knockdown induces apoptosis in UC cell line

The association of DPP4 expression and cell apoptosis was evaluated using flow cytometry. In contrast with control cells, J82 and RTCC-1 cells transfected with shDPP4 show significant induction of cellular apoptosis (Supplementary Figure S4). For J82 cells, the percentage of apoptotic cells increased from 3.97 ± 0.32% to 23.13 ± 0.55% after transfected with sh*DPP4* (*P* < 0.05). The RTCC-1 cells showed similar observation, which apoptotic cells increased from 0.74 ± 0.19% to 8.86 ± 0.42% after transfected with shDPP4 (*P* < 0.05).

## DISCUSSION

Albert Fischer first proposed in 1946 that the proteolytic ability of cancer cells could enhance tumor invasion through the degradation of the surrounding extracellular matrix [21]. Since then, a lot of efforts have been made in disclosing the mechanisms of various types of protease that positively or negatively affect the biological behavior cancers. It is now well established that pericellular protease plays an crucial role in the tumor microenvironment (TME) in enhancing or weakening the tumor invasiveness, tumor growth, angiogenesis, and inflammation [7]. Thus, a protease is expected to exert opposing functions in different tumors.

DPP4 family is the member of the serine peptidases family S9. The primary family members of DPP4 family S9B are DPP4, FAP, DPP8, and DPP9 [22]. Using data mining technique, we identified FAP and DPP4 transcripts were significantly expressed in UBUC. Although the log ratio of FAP transcript is higher than DPP4, our preliminary result shows that FAP has significantly associated with pT stage of UTUC and UBUC but not nodal metastasis. Unlike DPP4, univariate analysis showed that FAP expression was associated with poor DSS outcome only. The association of FAP expression and a malignant tumor was well studied in a various tumor. Overexpression of FAP is associated with poor outcome in some neoplasm (pancreatic, hepatocellular, and colonic malignancies) but not others (breast cancer). [23] It is likely that FAP expression affected the tumor biological behavior through remodeling of the cancer cell microenvironment and regulation of the infiltration of inflammatory cells.

DPP4 is a membrane-bound dimeric peptidase that ubiquitously expressed on various cell types, including lymphocytes, endothelial cells, and epithelial cells. The functions of DPP4 include adenosine deaminase (ADA) binding, serine peptidase activity, and ECM binding. DPP4 usually cleaved the N-terminal dipeptides from polypeptides with proline or alanine in the penultimate position [10]. Through the degradation of chemokines and cytokines, DPP4 functions as a regulator of inflammatory and immunological response, signal transduction, and apoptosis. Our results show that DPP4 knockdown cells have lower proliferative activity and enter G0/G1 phase more readily. This finding is in concordance with those observed by Jang et al. They showed in their results that after inhibiting DPP4 using vildagliptin, the proliferation of tumor cell was suppressed and the mitotic activity was halted [24]. It is likely that DPP4 regulate the tumor cell growth through generation of chemokines and cytokines, such as IL-6R [7]. Additionally, we disclosed that suppression of DPP4 expression in UCs cell line promote apoptosis of the cells. This observation is contradicted with the finding by Aoyama et al., who showed that alogliptin, a DPP4 inhibitor, attenuated thoracic aortic constriction (TAC) induced myocardial apoptosis [25]. Furthermore, linagliptin, another DPP4 inhibitor, was also shown to suppress the free fatty acid-bound albumin-induced apoptosis of renal proximal tubular cells [26]. This may because the DPP4 role in regulating apoptosis is different under the non-physiological state. Choi et al. show that DPP4 increase the expression of PIN1, which is a master regulator of mitochondrial apoptosis. PIN1 has both pro-apoptotic and anti-apoptotic activities, depending on the biological context and localization of the target proteins [27]. The detailed mechanism that DPP4 promote anti-apoptotic effect in UC warrant further studies.

Evidence shows that DPP4 plays important roles in regulating tumor cell adhesion, invasion, and cell cycle arrest [28]. DPP4 expression levels also correlate with tumor aggressiveness and invasiveness, and have been confirmed in various tumors, including renal cell carcinoma, melanoma, gynecological cancer, and hematological malignancy [29, 30]. In our study, patients with UC that express a high level of DPP4 are more likely to develop an advanced disease and have aggressive tumors. Lam et al. demonstrated that DPP4 expression is increased in colonic tumors, and higher expression was observed in tumors with higher TNM stage and with metastasis [31]. The expression of DPP4 is regulated by transcription factors such as SP-1, EGFR, and AP-1 factor NF-1 [32]. Interestingly, EGFR plays major roles in the tumorigenesis of both UC and colorectal cancer [33]. Many studies have revealed that gene expression of particular proteases may increase according to the oncogene activity [7]. However, the similar outcome does not apply to all malignancies. In endometrial carcinoma, there is an inverse correlation between DPP4 expression and tumor grading [28]. Overexpression of DPP4 also prolonged the survival of the patient with malignant pleural mesothelioma [34]. In ovarian carcinoma and mesothelioma, increase DPP4 expression can also improve cancer cells susceptibility to chemotherapy [37]. All these findings suggest that DPP4 has multiple functional roles and accounts for different carcinogenesis in various malignancies.

To metastasize, tumor cells must build up their capacity for local invasiveness and for penetrating local barriers. By doing so, the tumor cells usually need to change their shape and adhere to each other and the extracellular matrix [6]. Choi et al. demonstrated that DPP4 promoted epithelial cell transformation and tumor metastasis through enhancing MEK/ERK and JNK/c-Jun signaling and transcription factor E2F1 activity [27]. In our study, DPP4 knockdown UC cells have lower migration and invasion ability. This implicates that DPP4 can promote tumor cell migration and invasion. The previous study revealed that a protease complex that formed by DPP4 and separase has a gelatin-binding domain and may facilitate the degradation of local ECM. Fibronectin is frequently expressed in UC, especially in the advanced stages [35]. DPP4 can promote stromal invasion and metastasis through DPP4/fibronectin adhesion [36]. Furthermore, by binding to ADA, DPP4 can activate plasminogen and degrade the extracellular matrix, such as collagen type IV [37]. However, these effects may not observe in other tumors. Using DPP4 transfected ovarian cancer cells, Kajiyama et al. show that the invasiveness of transfected cells has decreased. It is likely that this effect is due to the downregulation of E-cadherin, SMA, and MMP-2 expression by DPP4 [38]. It is probable that, under different microenvironments, DPP4 has diverse roles in the carcinogenesis of various carcinomas.

The association of DPP4 with patient survival has been addressed widely in different carcinomas. Many studies have demonstrated that serum DPP4 is significantly lower in different tumors, including head and neck squamous cell carcinoma, colorectal cancer, stomach, and gynecological tumors. Javidroozi et al. show that not only is plasma DPP4 level lower in cancer patients, but the level also decreased further as the tumor stage advanced [39]. When patients with different cancers were grouped according to DPP4 levels, lower serum DPP4 levels were significantly associated with shorter survival. However, later study shows that serum CD26 does not correlate with any clinicopathological factors except Her2 positivity [40]. It has been suggested that T-lymphocytes are the major source of plasma DPP4, and the development of tumor-specific T-cell tolerance will decrease the serum DPP4 level [8]. Thus, the usefulness of serum CD26 in tumor screening and predicting outcome warrant further study. In our study, we demonstrated that UC overexpressing DPP4 is characterized by shorter DSS and MeFS. Similar observations have been described in some tumors, such as colorectal carcinomas and gastrointestinal stromal tumors, but not in others, such as mesotheliomas, prostate cancer, melanoma, and gynecological cancers [12, 31, 34, 41, 42]. Post-transcriptional modification may took part in determine the expression of DPP4. In melanocytes, study shows that lncRNA SPRIGHTLY down-regulated DPP4 gene expression [43]. The difference in lncRNA expression may be a reason for the difference in DPP4 expression [44].

Inflammatory cells are the major members of the tumor microenvironment. In UCs, increase infiltration of pro-tumor N2 neutrophils and CD204+ macrophage in tumor parenchyma will promote tumor invasion and are poor survival indicators [45–47]. CXCL10, a chemoattractant for immune cells such as T lymphocytes and monocytes, is a substrate of DPP4. Recently, a study shows that the inhibition of DPP4 will increase CD4+ and CD8+ T lymphocytes and delayed tumor growth. They concluded that presence of DPP4 would repress CXCR3-mediated anti-tumor immunity and thus limited the infiltration of T lymphocytes [48]. This could partially explain our results, as UC with higher levels of DPP4 frequently associated with high histological grading and advanced tumor pathological (pT) staging. Currently, various immunotherapies, clinical or preclinical, have been applied for the treatment of UC at aimed at improving anti-tumor immune response [49]. DPP4 inhibitor will be a good candidate to be included in the immunotherapies regimen for UCs treatment.

In conclusion, we observed that overexpression of DPP4 in UC correlated with destructive tumor behavior. Overexpression of DPP4 promotes tumor cell growth, proliferation, and enhance cell migration and invasion. Suppress DPP4 expression significantly attenuate UC aggressiveness and promote apoptosis in UC cells. Also, DPP4 is an independent prognosticatory biomarker in urothelial carcinoma. Further research to elucidate the mechanism of DPP4 contributed to the malignant behavior in urothelial carcinoma and the effectiveness of DPP4 inhibitors as targeted therapy are warranted.

## MATERIALS AND METHODS

### Data mining on the GEO to identify overexpressed transcripts in UBUCs

We identified two datasets from GEO, GSE32894 https://www.ncbi.nlm.nih.gov/geo/query/acc.cgi?acc=GSE32894 and GSE31684 ( http://www.ncbi.nlm.nih.gov/geo/query/acc.cgi?acc=GSE31684). The former dataset is composed of 308 UCs that analyzed with Illumina HumanHT-12 V3.0 expression beadchip and the later is generated from 93 UCs by using Affymetrix Human Genome U133 Plus 2.0 Array. Our analysis was specifically focused on genes that are classified into the functional category of proteolysis (GO:0006508). Transcripts with *p* < 0.001 and positive log2 -transformed fold change of expression were selected as candidates. Detailed of this procedure had been described in our previous work [50].

### Patients and tumor specimens

This study was approved by the Institutional Review Board of Chi Mei Medical Center (10302015). To evaluate the transcript expression of DPP4, 20 snap-frozen UBUC tumor samples were enrolled, including ten muscle-invasive (pT2-pT4) and ten non-muscle-invasive (pTa-T1). For the preliminary validation to identify the most significant gene among the candidates, a pilot batch of 60 UBUCs and 60 UTUCs were also enrolled. Lastly, the significance of DPP4 expression was analyzed in an independent cohort containing 635 well-characterized cases consecutively treated from 1996 to 2004. This cohort consists of 295 tumors that arose from the urinary bladder (UBUC) and 340 tumors that originated from the upper urinary tract (UTUC). The initial treatment for these patients was surgical intervention with curative intent. For patients with pT3 or pT4 stage UBUC, with or without nodal involvement, surgeries were followed by cisplatin-based adjuvant chemotherapy. In UTUC, only 29 of the 106 patients with pT3 or pT4 disease received adjuvant chemotherapy. Criteria for clinicopathological evaluation were essentially identical to that in our previous work [51]. Hematoxylin-eosin sections of all cases were reevaluated by two pathologists (CFL & IWC).

### Laser capture microdissection (LCM)

Detailed of this procedure had been described in our previous work [52]. Approximately 1500 tumor cells were dissected from each fresh sample using the Veritas automated LCM system (Arcturus Engineering, Mountain View, California, USA).

### RNA extraction and quantitative real-time RT-PCR

The collected UBUC tumor cells from LCM and the cultured cell lines were submitted for total RNAs extraction by using the RNeasy Mini Kit (QIAGEN). The extracted RNAs were subjected to reverse-transcription reactions using SuperScript III (Invitrogen) for cDNA synthesis. We used pre-designed TaqMan assay reagents coupled with ABI StepOnePlus System (Applied Biosystems) to measure *DPP4* (Hs00175210_ml) mRNA abundance. POLR2A (Hs01108291_m1) was used as the internal control. The fold of expression of *DPP4* relative to normal urothelium was calculated. The procedure is identical to that described in our previous work [53].

### Immunohistochemical staining, interpretation, and scoring of DDP4

The formalin-fixed paraffin embedded samples from the first cohort were assembled into recipient blocks of tissue microarrays (TMA) containing triplicate 1.5-mm tissue cores for each case [50–51]. Tissue sections of 4-μm thickness were prepared by standard procedure. After antigen retrieval, we incubated the sections with a primary antibody targeting DPP4 (1:100, Clone EPR5883(2), Epitomics) at a dilution of 1:100 for an hour. Primary antibodies were detected using the DAKO ChemMate EnVision Kit (K5007, Carpinteria, CA, USA). The presence of brown chromogen in the cytoplasm of tumor cells indicated positive immunoreactivity. A sample incubated without the primary antibody was used as a negative control.

The immunostained slides were blindly evaluated by two pathologists (PIL & CFL) without prior knowledge of clinical and follow-up data. A H-score of DPP4 immunoreactivity was assigned to each case by combining the percentage and the cytoplasmic intensity of the positively stained tumor cells. The equation is as follows, H-score = ΣPi (i + 1), where i is the intensity (0 to 3+), and Pi is the percentage of stained tumor cells for each intensity (0% to 100%). This equation originates a score ranging from 100–400, where 100 indicates 100% of tumor cells are negative, and 400 means 100% of tumor cells were strongly stained (3+) [54, 55].

### Cell culture and established stable DPP4 knockdown clones

Three human urothelial tumor cells lines, J82, TCCSUP, and RTCC-1, were included. J82 and TCCSUP were purchased from ATCC (Manassas, VA 20108, USA). RTCC-1, an urothelial carcinoma cell lines derived from renal pelvis, is a gift from Prof. Chiang LC [56]. A normal urothelial cell primary cell, HUC (ScienCell Research Laboratories, San Diego, CA), was used as a control. These cells were grown based on suggested medium and conditions and had been described in our previous work [57]. To generate DPP4 knockdown cells, we transfected J82 and RTCC-1 with the lentivirus that carries targeted short hairpin RNA sequence. We purchased the lentiviral expression plasmids from the National RNAi Core Facility, the Genomic Research Center of the Institute of Molecular Biology, Academia Sinica, Taiwan. The shRNA sequences in the lentiviral expres–sion vectors were pLKO.1-*shLacZ* (TRCN0000072223: 5′-TGTTCGC ATTATCCGAACCAT-3′), pLKO.1-*shDPP4*#1, TRCN0000050773: 5′- GCCCAATTTAACGACACA GAA-3′, pLKO.1-*shDPP4*#2, TRCN0000050774: 5′- CCAGAAGACAACCTTGACCAT-3′. The virus was produced as previously described [58]. Viral supernatants were harvested in the conditioned medium. J82 and RTCC-1 were plated in a 6-well plate at a density of 1 × 10^6^ per well and were incubated with viral supernatants for 48 hours.

### Western blot assays

The tumor cells were lysed with cell lysis buffer, and equal amounts of protein extract were separated by 4%–12% gradient NuPAGE gel (Invitrogen) and transferred onto polyvinylidene difluoride membranes (Amersham)[52]. The membranes were later probed with primary antibodies against proteins of interest at 4°C overnight and then incubated with the secondary antibody at room temperature for one hour. Primary antibodies used were as followings: DPP4 (1:5000, Epitomics) and GAPDH (1:10000, Chemicon).

### Cell cycle analysis with flow cytometry

We rinsed the harvested cells cultured on a 6 cm dishes with HBSS, fixed in ice-cold 70% ethanol, and stored at −20°C. J82 and RTCC-1 cells transfected with either *shLacZ* or *shDPP4*#1 which showed more pronounced growth inhibition were pelleted and re-suspended in PI/RNase Staining Buffer (BD Biosciences) before the analysis. They were stained in the dark for 15 minutes. The detection of the cell cycle was performed by using the flow cytometer (NovoCyteTM 2000, ACEA) and NovoExpressionTM software. Around 10,000 events were obtained during each analysis, and the proportions of cells in each cycle phase were calculated. Each experiment was repeated at least three times.

### 2,3-bis-(2-methoxy-4-nitro-5-sulfophenyl)-2H-tetrazolium-5-carboxanilide (XTT)-based assay

The XTT-based assay (Sigma, St. Louis, MO, USA) is carried out based on the product manual. In brief, we seeded the tumor cells in 96-well flat-bottom plates, at a density of 3,000–5000 cells per well, that contained phenol red-free medium for 48 h. We then will incubate the cells in a 37°C, humidified atmosphere that contained 5% CO2. After incubated for 24, 48, or 72 hours, we removed the culture medium and added 20 μl of XTT reaction solution to each well. After incubated for four hours, the optical density will be measured by using enzyme-linked-immunosorbent assay (ELISA) microplate reader (DAS Instruments, Rome, Italy) for absorbance at a wavelength of 450 nm against a reference wavelength of 630 nm.

### Cell migration and invasion assays

The migration assay was carried out by using the Falcon HTS FluoroBlok 24-well inserts (BD Biosciences) and the invasion assay by the 24-well Collagen-Based Cell Invasion Assay (ECM 554, Millipore). These tests were performed according to the manufacturer's instructions. In brief, an equal amount of cells were suspended in serum-free medium and were added to the upper chambers. The plates were then incubated at 37°C and 5% CO2 for six h to allow migration or invasion of the cells through the membrane into the lower chamber. After the non-invading cells in the upper chamber were removed, the migrated or invaded cells were stained, dissolved in buffer, and transferred to 96-well plates for colorimetric reading.

### Flow cytometric assays for cell apoptosis

Annexin V/propodium iodine (PI) staining coupled with flow cytometric analysis was performed to detect the percentage of apoptotic cells. A total of 10^5^ of J82 and RTCC-1 cells, transfected with either *shLacZ* or *shDPP4*#1 were plated for 24 h and then incubated with Annexin V-FITC kit (BD Pharmingen) that containing PI for 15 min. The cell percentages of cells at early apoptosis, late apoptosis, and necrosis were calculated from three independent experiments.

### Statistical analysis

Statistics were performed using SPSS V.12.0 software (SPSS Inc. Chicago, IL, USA). The median H-score of DPP4 dichotomized the study cohort into high expression and low expression groups. The associations between DPP4 expression status and various clinicopathological parameters were evaluated using the chi-square test. The endpoints for statistical analysis were disease-specific survival (DSS) and metastasis-free survival (MeFS), calculated from the starting date of surgery to the date the event developed. Patients lost to follow-up were censored at the latest follow-up date. We compared the expression levels of DPP4 mRNA between the early stage (pTa-pT1) and advanced stage (pT2-pT4) by using Mann-Whitney *U* test. Survival curves were plotted using the Kaplan-Meier method, and the prognostic differences between groups were evaluated using the log-rank test. Parameters demonstrating *p*-values less than 0.1 in the univariate analysis were enrolled into the multivariate test, which was carried out using the Cox proportional hazards model. For all analyses, two-sided tests of significance were used with *p* < 0.05 considered significant.

### Abbreviations

Dipeptidyl peptidase 4 (DPP4), Urothelial carcinoma (UC), urinary bladder urothelial carcinoma (UBUC), urinary tract urothelial carcinoma (UTUC), disease-specific survival (DSS), metastasis-free survival (MeFS), Reverse transcription polymerase chain reaction (RT-PCR).

## SUPPLEMENTARY MATERIALS FIGURES AND TABLES


