# Supplementary material for: DPP4/CD26 overexpression in urothelial carcinoma confers an independent prognostic impact and correlates with intrinsic biological aggressiveness
Source: Oncotarget. 2016 Dec 7;8(2):2995–3008. doi: 10.18632/oncotarget.13820 (PMC5356858; doi:10.18632/oncotarget.13820)
Supplement: Supplementary file 1 [file oncotarget-08-2995-s001.pdf]

DPP4/CD26 overexpression in urothelial carcinoma confers an independent prognostic impact and correlates with intrinsic biological aggressiveness

Supplementary Materials

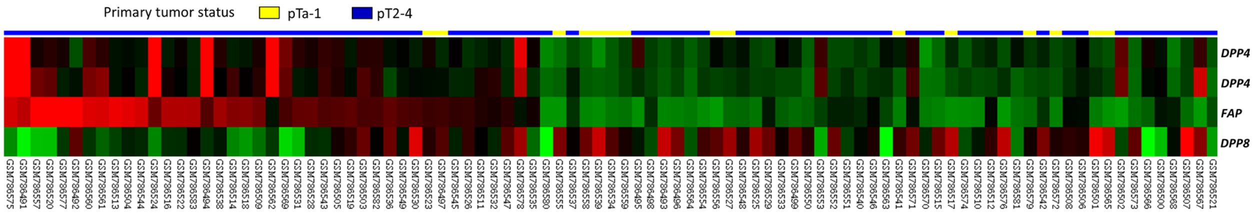

Supplementary Figure S1: Data mining on GSE31684 (GEO Omnibus) dataset identified three transcripts (4 probes) that were significantly associated with proteolysis (GO: 0006508) in urothelial carcinoma of urinary bladder (UBUC) The heat map shows significantly upregulated *DPP4* and *FAP* transcripts levels in muscle-invasive UBUC (pT2-pT4, *n* = 73). *DPP8*, on the other hand, is inversely associated with the pT stage. Low expression values are green, progression into dark and reds for higher values.

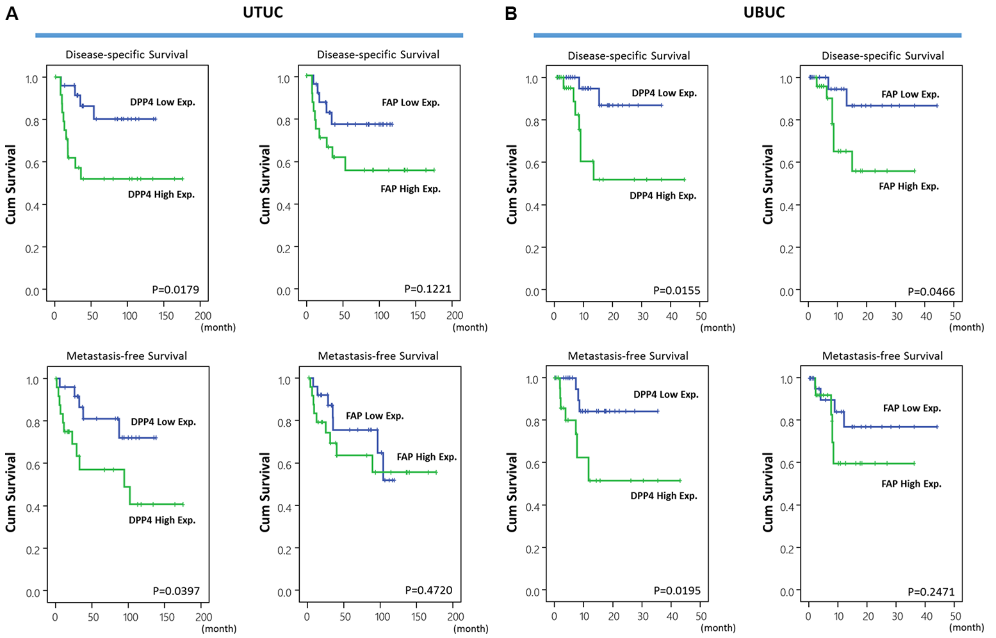

Supplementary Figure S2: A preliminary test for clinical significance of DPP4 and FAP by using immunohistochemistry in our pilot batch of cases. (**A**) The expression of DPP4 and FAP proteins were tested in a pilot batch of urothelial carcinoma of the upper urinary tract (UTUC, *n* = 60). The DPP4 immunoreactivity is significantly associated with disease-specific survival (DSS, *right upper* panel) and metastatic-free survival (MeFS, *right lower* panel). On the other hand, FAP expression is not associated with DSS (*left upper* panel) and MeFS (*left lower* panel). (**B**) The expression of DPP4 and FAP proteins were also tested in a pilot batch of UBUC (*n* = 60). The finding is similar, except FAP expression is significantly associated with DSS of UBUC (*p* = 0.0466).

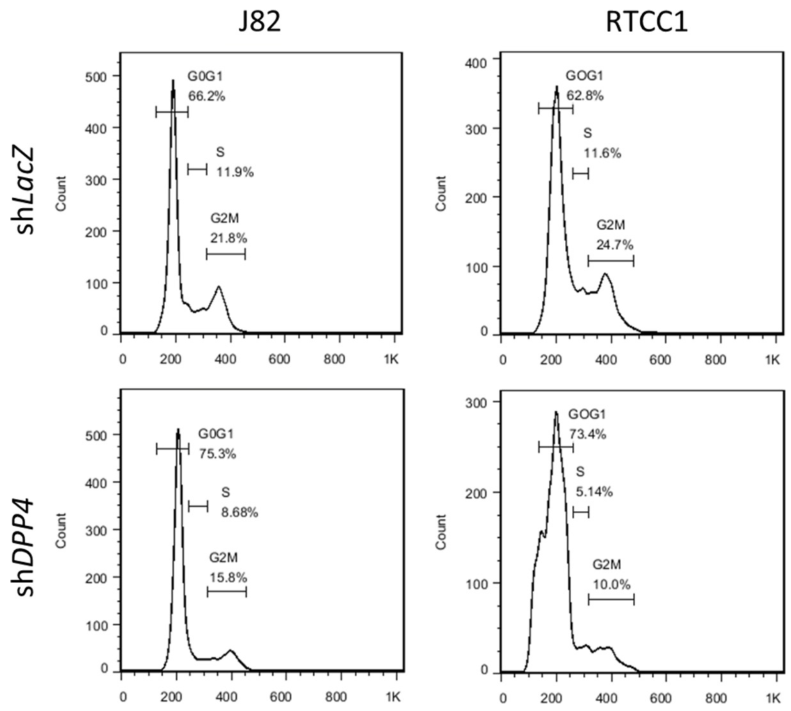

Supplementary Figure S3: DPP4 knockdown leads to a G0/G1 arrest. Depletion of DPP4 expression results in a significant G0/G1 accumulation of both J82 (*right* panel) and RTCC-1 (*left* panel) cells.

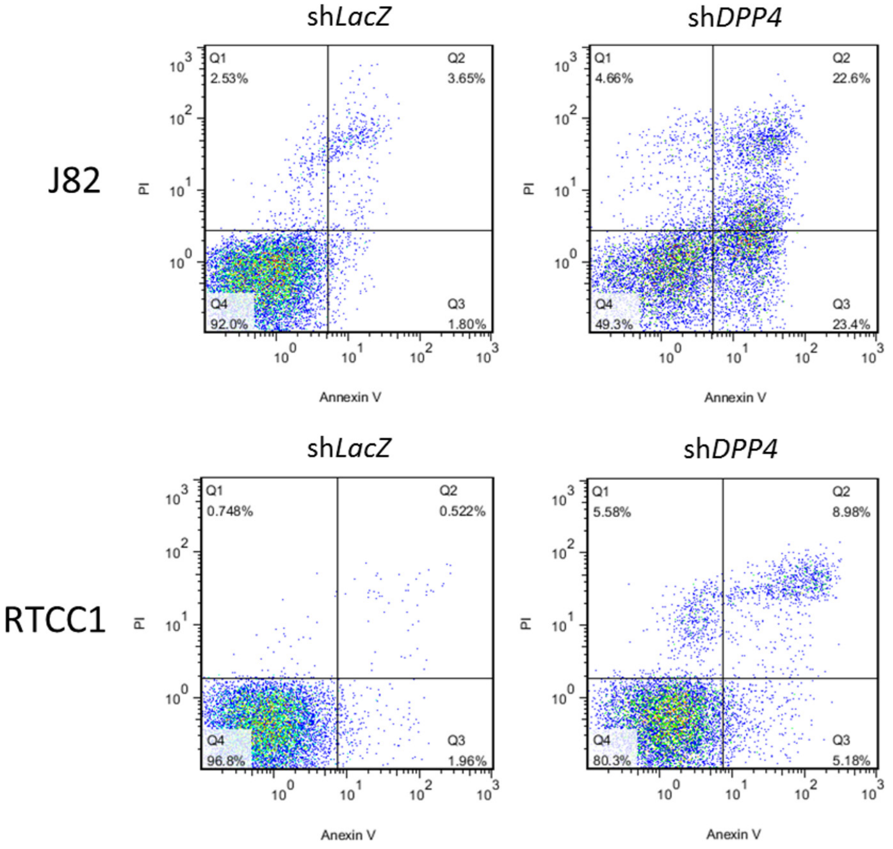

Supplementary Figure S4: DPP4 knockdown promote apoptosis of UC cell. Using Annexin V/propidium iodine (PI) stains, the flow cytometric assays demonstrated that J82 and RTCC-1 cell transfected with sh*DPP4* have higher amount of apoptotic cells (Annexin V +/PI +), in contrast with the control.

Supplementary Table S1: Summary of differentially expressed genes associated with *dipeptidyl-peptidase 4* activity and showed stepwise alterations during cancer progression in the transcriptome of urothelial carcinoma of urinary bladder (GSE32894)

Probe	Comparing T2-4 to Ta-T1		Gene Symbol	Biological Process	Molecular Function
	log ratio	*p*-value			
ILMN_1689160	−0.2952	0.0063	***DPEP2***	proteolysis	dipeptidase activity, dipeptidyl-peptidase activity, metal ion binding, metalloexopeptidase activity, peptidase activity, zinc ion binding
ILMN_1692535	0.495	< 0.0001	***DPP4***	T cell activation, proteolysis, regulation of cell-cell adhesion mediated by integrin, response to hypoxia	aminopeptidase activity, dipeptidyl-peptidase activity, peptidase activity, protein binding, protein homodimerization activity, serine-type endopeptidase activity
ILMN_1759801	−0.2349	< 0.0001	***DPP8***	immune response, proteolysis	aminopeptidase activity, dipeptidyl-peptidase activity, peptidase activity, serine-type endopeptidase activity
ILMN_2232854	0.976	< 0.0001	***FAP***	proteolysis	dipeptidyl-peptidase activity, metalloendopeptidase activity, peptidase activity, protein homodimerization activity, serine-type endopeptidase activity
ILMN_2312732	−0.2454	< 0.0001	***DPP8***	immune response, proteolysis	aminopeptidase activity, dipeptidyl-peptidase activity, peptidase activity, serine-type endopeptidase activity

Supplementry Table S2: Summary of differentially expressed genes associated with *dipeptidyl-peptidase 4* activity and showed stepwise alterations during cancer progression in the transcriptome of urothelial carcinoma of urinary bladder (GSE31684)

Probe	Comparing T2-4 to Ta-T1		Gene Symbol	Biological Process	Molecular Function
	log ratio	*p*-value			
203716_s_at	0.3656	0.002	***DPP4***	immune response, proteolysis	aminopeptidase activity, dipeptidyl-peptidase IV activity, hydrolase activity, peptidase activity, prolyl oligopeptidase activity, serine-type endopeptidase activity, serine-type peptidase activity
209955_s_at	1.5836	0	***FAP***	proteolysis	dipeptidyl-peptidase IV activity, hydrolase activity, metalloendopeptidase activity, peptidase activity, prolyl oligopeptidase activity, protein dimerization activity, protein homodimerization activity, serine-type endopeptidase activity, serine-type peptidase activity
211478_s_at	0.5544	0.0004	***DPP4***	immune response, proteolysis	aminopeptidase activity, dipeptidyl-peptidase IV activity, hydrolase activity, peptidase activity, prolyl oligopeptidase activity, serine-type endopeptidase activity, serine-type peptidase activity
220939_s_at	-0.5485	0.0021	***DPP8***	immune response, proteolysis	aminopeptidase activity, dipeptidyl-peptidase IV activity, dipeptidyl-peptidase activity, hydrolase activity, peptidase activity, serine-type endopeptidase activity, serine-type peptidase activity

Supplementary Table S3: Correlations between DPP4 and FAP Expression and other important clinicopathological parameters in urothelial carcinomas.

Parameter	Category	Urothelial Cancer of upper urinary tract							Urothelial cancer of urinary bladder						
		Case No.	DPP4 Exp.		*p*-value	FAP Exp.		*p*-value	Case No.	DPP4 Exp.		*p*-value	FAP Exp.		*p*-value
			Low	High		Low	High			Low	High		Low	High	
**Primary tumor (T)**	Ta-T1	16	13	3	<0.001*	12	4	0.048*	12	11	1	0.004*	10	2	0.018*
	T1	9	7	2		4	5		7	2	5		4	3	
	T2-T4	25	5	20		9	16		31	12	9		11	20	
**Nodal metastasis (N)**	Negative (N0)	46	25	21	0.037*	24	22	0.297	43	23	20	0.221	22	21	0.684
	Positive (N1-N2)	4	0	4		1	3		7	2	5		3	4	

***Statistically significant.**

Supplementary Table S4: Univariate log-rank and multivariate analyses for disease-specific and metastasis-free survivals in upper urinary tract urothelial carcinoma

Parameter	Category	Case No.	Urothelial Cancer of upper urinary tract				Urothelial cancer of urinary bladder			
			Disease-specific survival		Metastasis-free survival		Disease-specific survival		Metastasis-free survival	
			No. of event	*p*-value	No. of event	*p*-value	No. of event	*p*-value	No. of event	*p*-value
**DPP4 Exp.**	Low	25	4	**0.0179***	5	**0.0397***	2	**0.0155***	3	**0.0195***
	High	25	11		11		7		8	
**FAP Exp.**	Low	25	5	0.1221	7	0.4720	2	**0.0466***	4	0.2471
	High	25	10		9		7		7	

***Statistically significant.**

Supplementary Table S5: Correlations between DPP4 Expression and other important clinicopathological parameters in urothelial carcinomas

Parameter	Category	Upper Urinary Tract Urothelial Carcinoma				Urinary Bladder Urothelial Carcinoma			
		Case No.	DPP4 Expression		*p* -value	Case No.	DPP4 Expression		*p*-value
			Low	High			Low	High	
Gender	Male	158	74	84	0.277	216	104	112	0.339
	Female	182	96	86		79	43	36	
Age (years)	< 65	138	72	66	0.508	121	58	63	0.587
	≥ 65	202	98	104		174	89	85	
Tumor location	Renal pelvis	141	71	70	0.110	-	-	-	-
	Ureter	150	81	69		-	-	-	-
	Renal pelvis & ureter	49	18	31		-	-	-	-
Multifocality	Single	278	145	133	0.092	-	-	-	-
	Multifocal	62	25	37		-	-	-	-
Primary tumor (T)	Ta	89	62	27	**< 0.001***	84	56	28	**< 0.001***
	T1	92	64	28		88	50	38	
	T2-T4	159	44	115		123	41	82	
Nodal metastasis	Negative (N0)	312	165	147	**< 0.001***	266	138	128	**0.033***
	Positive (N1-N2)	28	5	23		29	9	20	
Histological grade	Low grade	56	36	20	**0.019***	56	33	23	0.130
	High grade	284	134	150		239	114	125	
Vascular invasion	Absent	234	145	89	**< 0.001***	246	137	109	**< 0.001***
	Present	106	25	81		49	10	39	
Perineural invasion	Absent	321	164	157	0.098	275	142	133	**0.021***
	Present	19	6	13		20	5	15	
Mitotic rate (per 10 high power fields)	< 10	173	100	73	**0.003***	139	74	65	0.269
	>= 10	167	70	97		156	73	83	

*** Statistically significant.**
